# Ischemic Infarct of the Hand Knob Gyrus: Natural History, Morphology, and Localizing Value of the Omega Sulcus – A Case Report With a Side Note on the Dynamic Forces Underlying Sulci Formation

**DOI:** 10.7759/cureus.9024

**Published:** 2020-07-06

**Authors:** Hassan Kesserwani

**Affiliations:** 1 Neurology, Flowers Medical Group, Dothan, USA

**Keywords:** ischemic cva, neural sulcus, embolic cva

## Abstract

We describe a case of right-hand weakness localizable to an ischemic infarct of the hand knob gyrus of the left primary motor cortex. The hand knob gyrus is usually delimited by an omega-shaped sulcus, which is useful in outlining the posterior border of the frontal lobe. Being a cortical infarct, a potential embolic etiology was investigated and found, in keeping with previous case series reports. This finding, along with the relatively benign nature of this type of ischemic infarction, is emphasized. Identifying the omega sulcus is important as it is a reliable landmark demarcating eloquent motor cortex, which is critically important in planning surgical removal of brain tumors. In this case report, we review the largest case series of ischemic infarcts of the hand knob gyrus. We also describe the morphology of the omega sulcus, discuss the various theories underlying the convolutions or folding of the gyri of mammalian and human brains, and speculate as to why ischemic infarcts of the hand knob gyrus carry a relatively benign outcome.

## Introduction

In this report, we describe a case of right-hand weakness localizable to an ischemic infarct of the hand knob gyrus of the left primary motor cortex, the precentral gyrus. The hand knob gyrus is delimited by an omega-shaped sulcus, which is clinically useful in localizing the posterior border of the frontal lobe. Fissuration of the brain with its undulating gyri and sulci has a rich history in neurology and paleoneurology. We describe in detail an ischemic infarction of the hand knob gyrus and its clinical course, succinctly review the largest case series reports, and discuss the mechanistic forces underlying the folding of gyri. This latter field of study is contentious with various competing theories to explain cortical folding, such as tension along radial axons, tension along tangential axons, and intrinsic curvature of nervous tissue modulated by differential growth. We address these mechanistic forces and finally speculate on why infarcts of the hand knob gyrus carry a relatively benign prognosis.

The precentral gyrus runs on the lateral surface of the frontal lobe, anterior to the central sulcus. The primary motor cortex lies in the precentral gyrus and controls voluntary motion. The corticospinal tract, corticobulbar tract, and cortico-rubro-spinal tract originate within the precentral gyrus. The hand knob gyrus is located on the precentral gyrus. It was described by a functional MRI study in 1997 as a region shaped like an omega or epsilon [1]. A lesion here is associated with finger or hand weakness. This gyrus is also useful for identifying the precentral gyrus directly as it outlines the middle knee of the central sulcus. Ischemic lesions here are cortical and likely embolic, necessitating the exclusion of atrial fibrillation, or a lesion more proximally in the carotid arteries or aortic arch, or other cardiac sources. Hence, it is a clinically useful localizing sign.

Fissuration of the mammalian brain has played a revolutionary and insightful role in helping us understand functional neuroanatomy and human brain paleoneurology [2]. Examples include the controversial lunate sulcus, the archaic rhinal sulcus, and the historically important sulci demarcating Broca's cap, the third inferior frontal convolution, to mention a few. But an outline of this exciting topic is beyond the scope of this discussion. Van Essen has theorized that a morphogenetic process involving axons, dendrites, and glial processes explains the convolutions of mammalian cortices, by approximating adjacent gyri through mechanical tension [3]. In the cerebral cortex, tension along axons of the white matter induces folding. In the cerebellum, tension along parallel fibers elongates the axons and folds them like an accordion. By minimizing the sum-total length of axonal and dendritic wiring, the length of the circuitry of the brain is kept compact. However, this dynamic theory of axonal tension has not been borne out by studies in ferret cortices [4]. Instead, we will focus on the intrinsic curvature of nervous tissue. This theory is borne out by differential geometry and measurement of surrogate markers such as the intrinsic curvature of the cortical surface [5]. But the study of the actual mechanisms of fissuration of the mammalian brain is still a hotly debated and researched topic.

We present this case as a great example of brain localization to a specific motor function: hand and finger power. Raymond Adams had emphasized the importance of seeking an embolic source for ischemic infarcts of cortical gyri [6]. In our case, the whole arterial tree was interrogated from the cardiac chambers and aortic arch via transesophageal echocardiogram (TEE) to the middle cerebral artery via transcranial Doppler. A moderately sized atrial shunt was discovered as the most proximate etiology in a middle-aged man with low risk for cerebral infarction. We outline the case in detail and then segue into the morphology of the omega sulcus, propose reasons for the relatively benign nature of this type of ischemic infarct, and then briefly overview the mechanical forces underlying cortical folding.

## Case presentation

We describe the case of a healthy 51-year-old right-handed man who, while preparing a cup of coffee in the morning, had noted heaviness of his right hand and forearm. He had struggled to fix his breakfast but dismissed it as a "funny" arm caused by sleeping on it. While tying his shoes, he had had to rely more on his left hand as he had diminished deftness with his right hand and fingers. He had noted some tingling of his right fingertips, but this had been brief and only lasted for a few minutes. He had stoically gone to work and noted that his symptoms had completely resolved within six hours. He denied any slurring of speech, language issues, or visual changes. He had smoked 10 cigarettes a day for 31 years. He led a healthy lifestyle, exercised frequently, and received an annual medical check-up. His diet was balanced and mixed. He denied any family history of premature heart attacks or strokes. He denied alcohol use. His medications included buspirone for anxiety.

On examination, his height measured 6 feet and 2 inches; his weight was 188 pounds and BMI was 24.1. His office blood pressure was 119/80 mmHg, and he had a regular pulse rate of 67 bpm. Auscultation of the neck was free of a carotid bruit. No audible murmur was noted on auscultation of the precordium. His neurological examination was entirely normal. Specifically, his posture, gait, and stability were entirely normal. He was able to tandem fluently and stand on either foot with ease. Cranial nerve examination was entirely normal with no evidence of an ophthalmoplegia or facial weakness. Visual fields were full to confrontation. His speech with repetition was entirely normal. Visual praxis was preserved with simple pantomime, and optokinetic nystagmus was symmetric with a horizontally striped cloth.

Motor examination of upper extremities revealed normal tone and no pronator drift. The sequence motion of fingers was entirely normal. Attention to praxis revealed normal ideomotor and ideational praxis, by applying rotatory deftness with a coin and transitive pantomime respectively. No dysmetria or kinetic tremor was noted with the arms. He was able to squat and rise with both knees, stand on his heels and toes, and wiggle his toes symmetrically. Reflexes were lively throughout the upper and lower extremities. Cortical sensory examination with stereognosis and graphesthesia was preserved in both hands.

**Figure 1:**
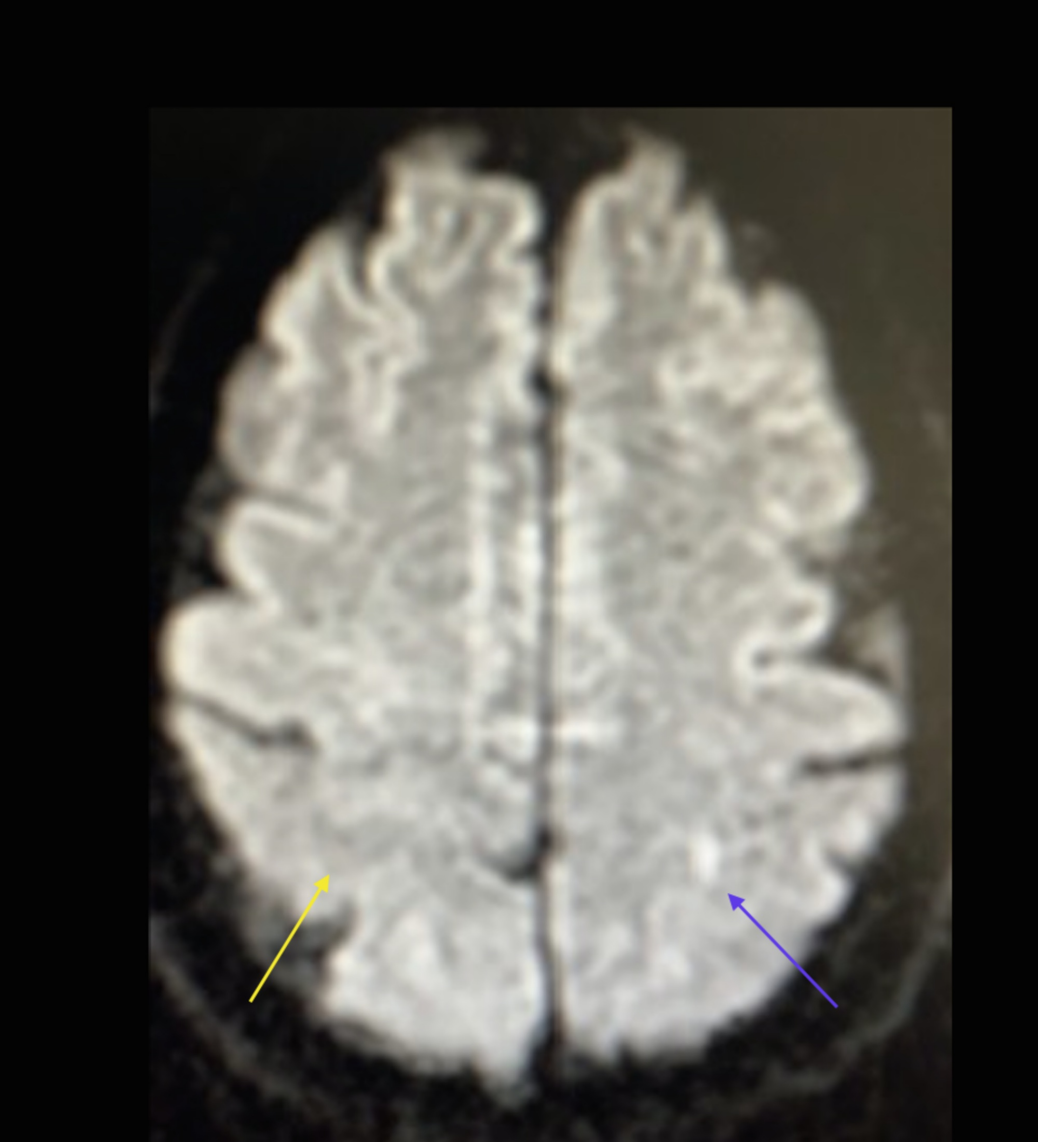
Diffusion-weighted sequence: 0.3 tesla MRI The image shows a faint outline of right hemisphere omega sulcus (yellow arrow) and left-hand knob gyrus ischemic infarct with obscuring of omega sulcus (blue arrow); the resolution was limited by the strength of the magnet MRI: magnetic resonance imaging

**Figure 2:**
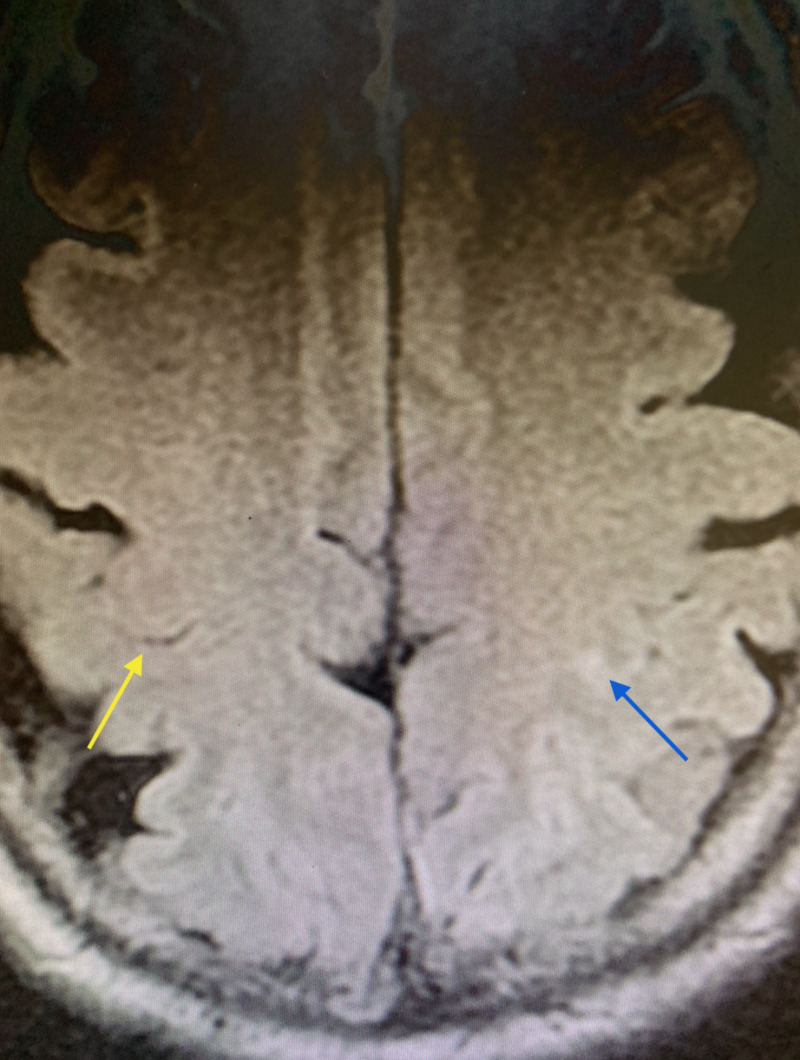
FLAIR sequence: 0.3 tesla MRI Right omega sulcus (yellow arrow), hyperintensity left-hand knob gyrus obscuring omega sulcus (blue arrow) MRI: magnetic resonance imaging; FLAIR: fluid-attenuated inversion recovery

A 0.3 tesla MRI study revealed an ischemic infarct involving the hand knob region of the motor strip of the left frontal lobe immediately adjacent to the omega sulcus. This was visible on the diffusion-weighted imaging sequence and fluid-attenuated inversion recovery (FLAIR) sequence as a hyperintensity (Figures 1, 2).

Considering the cortical infarct, and given that the patient was relatively young, he was screened for the antiphospholipid antibody syndrome, including lupus anticoagulant, immunoglobulin M (IgM) and immunoglobulin G (IgG) anti-phospholipid antibodies, and beta 2 glycoprotein, all of which came back normal. A cervical color carotid duplex scan revealed normal peak systolic velocities. A transcranial Doppler with a 2 MHz crystal with insonation of both temporal windows [middle cerebral arteries (MCA), MCA bifurcation, anterior and posterior cerebral arteries], sub-occipital window (both vertebral arteries and basilar artery), and trans-orbital windows (carotid siphon and ophthalmic arteries) revealed normal mean systolic velocities and pulsatility indices with monophasic waveforms and appropriate direction flows with a butterfly pattern of MCA bifurcation. TEE revealed a normal aortic arch, with a moderate right-to-left atrial shunt with agitated saline but no atrial septal aneurysm. Due to shunting and potential micro-emboli on the venous side of the circulation, a thrombophilia panel [protein C, protein S, antithrombin III activity, activated protein C (APC) resistance, prothrombin gene 20210 A mutation, factor VIII activity] was obtained and values were all normal. A 30-day event monitor showed no atrial fibrillation, and a loop monitor was implanted to rule out paroxysmal atrial fibrillation. Fasting blood lipids including fasting cholesterol, high-density lipoprotein (HDL), low-density lipoprotein (LDL), and total cholesterol/HDL ratios were normal. He was placed on baby aspirin 81 mg per day and was referred for shunt closure, as the right-to-left atrial shunt was the most proximate etiology for this ischemic infarct of the hand knob gyrus.

## Discussion

**Figure 3:**
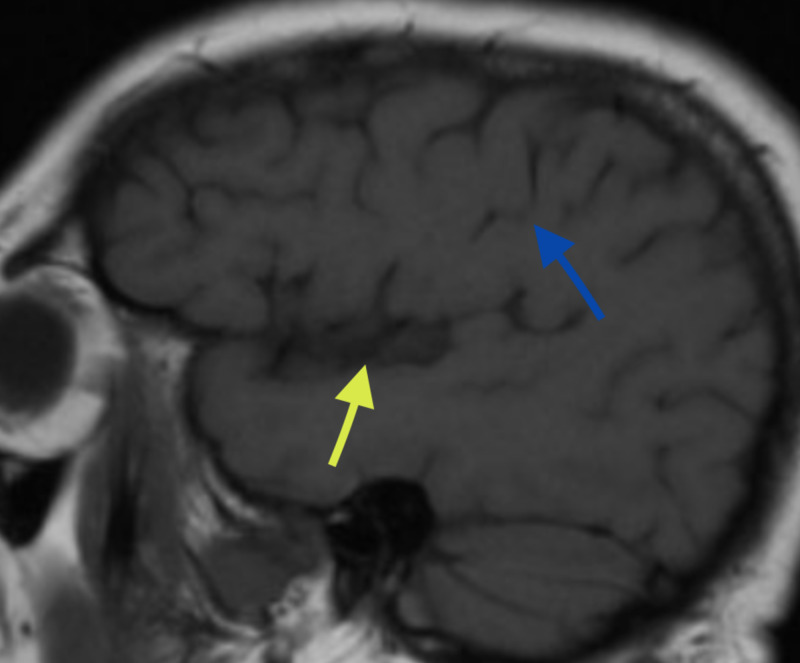
T1-weighted coronal MRI The image shows insula (yellow arrow) hook of omega sulcus on coronal view (blue arrow) MRI: magnetic resonance imaging

The central sulcus is usually sinusoidal with three curves: superior, middle, and inferior genu; with anterior, posterior, and anterior concavities respectively. The middle genu is shaped like an inverted omega and delimits the hand knob gyrus, so named because it is knob-shaped. The knob-like morphology is demarcated in 90% of the cases by an inverted omega and by a horizontal epsilon in 10% of the cases. In the sagittal plane, the hand knob gyrus appears to be hook-shaped. The mean length of this sulcus is 1.4 cm, and its mean separation from the midline is 2.3 cm [7]. On the sagittal section, the hook-shaped sulcus can be traced by identifying the insula and traveling backward with an imaginary line and then dropping a line vertically from the base of the hook so that the two lines meet at right angles (Figure 3).

**Figure 4:**
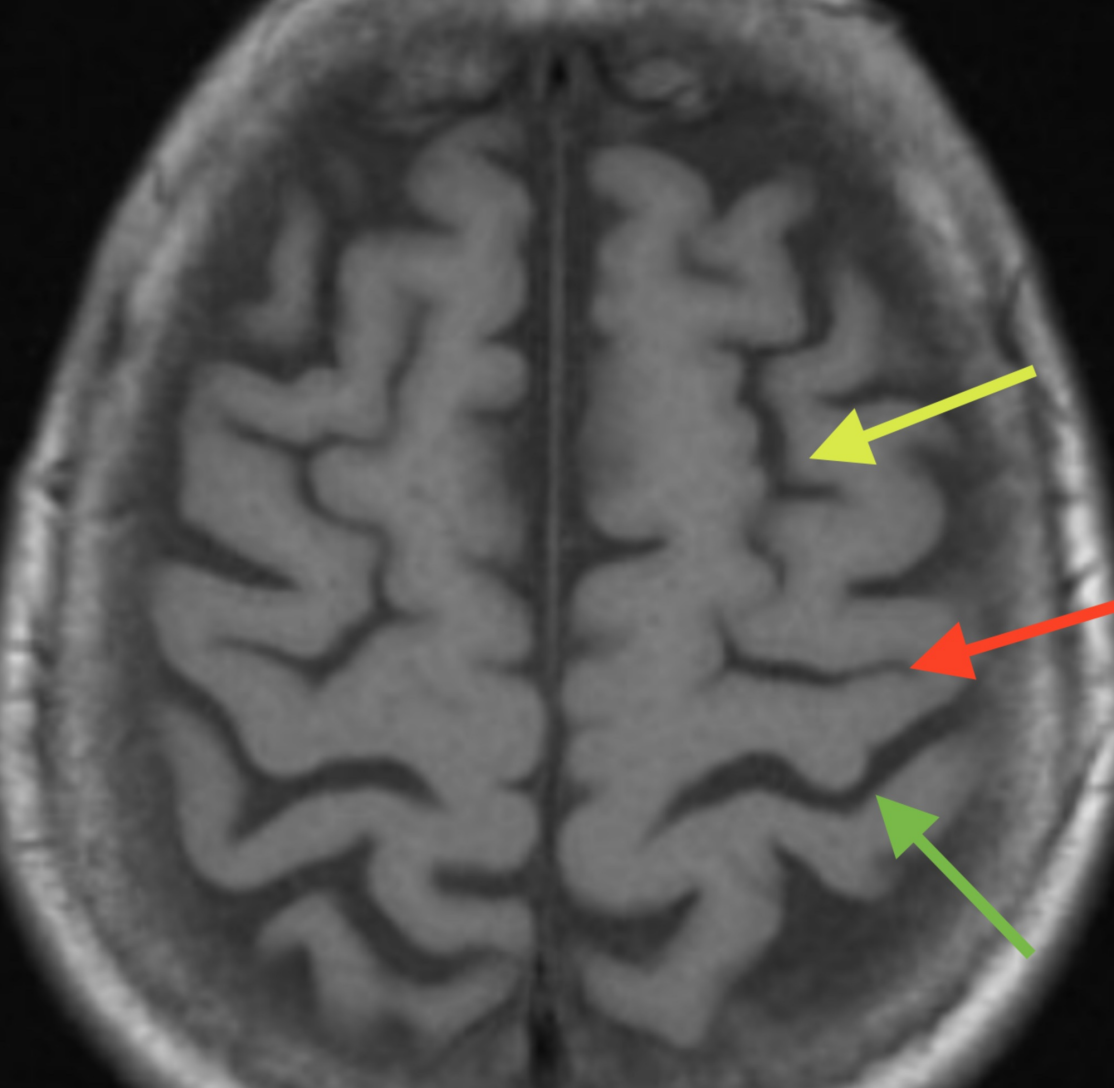
T1-weighted axial MRI The image shows superior frontal sulcus (yellow arrow), central sulcus (red arrow), and omega sulcus (green arrow) MRI: magnetic resonance imaging

In the axial plane, the omega sulcus has been documented in 100% of the hemisphere, rendering it a highly remarkable landmark, especially when compared to the superior frontal sulcus, which is continuous in up to 40% of hemispheres and may not meet the precentral sulcus in 8% of the cases. Intraoperatively, the hand knob area can be identified with ease by simply following the superior frontal sulcus backward where it meets the central sulcus; the knob is immediately behind, hence allowing the neurosurgeon to easily identify the hand motor cortex (Figure 4).

An ischemic infarct of the hand knob area can mimic a pseudo-peripheral nerve palsy as it may produce isolated weakness of the fingers, the lateral radial fingers, or the medial ulnar fingers. The ulnar fingers are represented medially and the radial fingers are represented laterally in the hand knob gyrus [8]. An MRI morphologic study of hand knob gyrus in 257 right-handed individuals demonstrated three additional variants of the delimiting sulcus of the hand knob gyrus: a medially asymmetric epsilon (2.9% of cases), a laterally asymmetric epsilon (7% of cases), and a null variant (1.8% of cases). No sexual dimorphism was noted [9].

In a case series of 17 subjects, up to 79% of the patients with ischemic infarcts were felt to have an embolic etiology, and it was confirmed in 50% of the cases. The prognosis was generally good to excellent in 77% of the patients by two years. This case series covered a time span of five years involving 866 ischemic strokes at this institution, providing an ischemic stroke rate of 1.5% involving the hand knob gyrus [10]. In another case series of 11 subjects collected over a three-year period from 339 ischemic strokes, the most common etiology was cryptogenic, namely embolic stroke of undetermined cause (seven patients), and two definite cardioembolic ischemic strokes [11]. Again, the prognosis was excellent. In another case series of 25 patients, the authors emphasized the fact that an ischemic stroke of the hand knob area may mimic peripheral nerve damage as the weakness may involve the lateral radial fingers, medial ulnar fingers, or asymmetric weakness of both. Again, an embolic etiology was emphasized and the natural history was mostly benign; 36% of the patients had greater than 50% stenosis of the ipsilateral internal carotid artery and 12% had atrial fibrillation [12].

The relatively benign course of ischemic infarcts to the hand knob gyrus may be attributed to the consilience of several anatomical facts. The precentral gyrus is supplied by both the anterior cerebral artery (ACA) and the MCA. The superior division of the MCA supplies the more lateral surface of the precentral gyrus, while the ACA supplies the medial portion. This anastomotic connection provides collateral flow, which may expedite recovery following occlusion of the MCA or a branch of the MCA. The ulnar fingers are supplied by the anastomosis and the radial fingers by distal branches of the MCA. This more than likely explains the asymmetric weakness of the fingers in some patients.

In a remarkable study of intra-cortical micro-electrode array recordings in quadriparetic patients, with recordings from the hand knob gyrus, strong neural tuning was recorded for a variety of face, leg, and arm movements, implying mixed representation in one area of the precentral gyrus. This is in addition to the well-known motor somatotopy, the homunculus. This provides great insight into the extent of neuroplasticity of the motor cortex. Hence, we can hypothesize that when a small region is infarcted, other areas of the precentral gyrus may compensate through whole-body tuning and neural encoding [13].

Gyrification (buckling and fissuring) is not a random process as many sulci are quite consistent within and across species, such as the primary sulci; the central fissure, and lateral fissure. Van Essen has postulated a mechanism of tension along axons pulling the gyri together [3]. However, the fact that the axons run parallel to the gyri, and that the tensions as demonstrated in ferrets are not strong enough to account for the buckling and the cortico-cortical association fibers emerging after the genesis of the primary sulci, challenges this hypothesis. An attractive hypothesis suggests that tangential surface expansion generates pressures that induce convolutions, and that this buckling reduces the expanding pressure [14]. However, this would be a stochastic process and does not explain the consistency of sulci. The consistency of sulci suggests an underlying genetic basis. Any theory would have to include the consistency of sulci, an underlying genetic basis, and a sound dynamical physical principle. However, a differential cortical expansion model with stress lines is a plausible mechanism, since we know that the cerebral cortex is heterogeneous (allocortex, paleocortex, and archicortex) with differential layering and spacing of neurons. Furthermore, there is a temporal rostrocaudal gradient of early embryonic cortical neural growth, and even the cortical progenitor cells are located in the regions of greatest tangential expansion. These factors provide a genetic template with stress lines that fold under geometric constraints.

An excellent surrogate marker to represent the folding of the cortex arises from the mathematical concept of intrinsic curvature. The intrinsic curvature is a fixed property of the surface, independent of the embedding manifold, of the three-dimensional space. It is also known as the Gaussian curvature. The intrinsic curvature does not change if one bends the surface without stretching. It is a remarkable result of differential geometry and is known as the Theorema Egregium, the most wonderful theorem, attributed to the great mathematician Carl Friedrich Gauss. The intrinsic curvature is calculated by computing the second fundamental form or Hessian of the surface. This determinant captures the curvature of a surface by calculating the second derivative of the surface normal unit vector [13]. Using computer software, the Gaussian curvature was computed for 65 individuals [5]. The results were impressive. The Gaussian curvature was different for different regions of the brain. For example, the parietal lobes and frontal surfaces had higher Gaussian curvatures than the cingulate and insular cortices. The occipital and temporal lobes values were between those of the latter two groups. The Gaussian curvature also correlated with another marker of curvature, the local gyrification index, which is a ratio of the total surface area of a region compared to a reference surface. On the other hand, although the extrinsic curvature reflects how a surface is embedded in a three-dimensional space, it does not influence the intrinsic curvature of a surface. For example, an expanding sphere remains an expanding sphere, since the intrinsic Gaussian curvature of the surface does not change. If we stretch the sphere at one small patch and produce a dumbbell, the Gaussian curvature changes, and the extrinsic curvature also changes. The converse is not true. That is, the extrinsic curvature does not change the metric or intrinsic curvature. For example, if we intersect the sphere with a plane, the intrinsic or Gaussian curvature of the circle created by the plane does not change.

These ideas are intuitive and easily proven mathematically [15]. Hence, the hypothesis that variable neuronal densities in different regions of the cortex lead to differential expansion and folding seems plausible. The neuronal density in different parts of the brain and in different layers of the cortex is genetically determined. Therefore, gyrification is a function of variable neuronal density and differential expansion and is a property of mammalian cortices. The folding of the cortex is an intrinsic geometrical property of the surface, just as the plasma membrane may fold into cells or soap bubbles and expand into spheres. Here we have a hypothesis with a solid mathematical foundation, good surrogate markers, and an underlying genetic basis. Therefore, cortical folding or gyrification can be explained by the intrinsic morphological properties of a surface as the driving force for the intrinsic Gaussian curvature, leading to the convolution of the cortex. Further research including mathematical modeling, advanced neuroimaging data, and developmental neuroembryological studies are needed to confirm and build on this exciting data.

## Conclusions

Our case confirms the prevailing ideas on ischemic infarcts of the hand knob gyrus, namely their frequent embolic etiology and their relatively benign nature. However, we further explored the morphology, geometry, and localization of the omega sulcus and hypothesized as to why ischemic infarcts of this hand knob gyrus are relatively benign, by adumbrating on the neuroplasticity of the primary motor cortex and the rich anastomotic blood supply of the hand knob gyrus. We also analyzed the dynamic forces underlying gyrification of the human and mammalian cortices and highlighted the latest ideas regarding the intrinsic curvature of differentially expanding neural tissue. We believe these ideas have far-reaching consequences in understanding the all-important phenomenon of fissuration of the human and mammalian brains.
